# Epidemiology of *Plasmodium vivax* Malaria in India

**DOI:** 10.4269/ajtmh.16-0163

**Published:** 2016-12-28

**Authors:** Anupkumar R. Anvikar, Naman Shah, Akshay C. Dhariwal, Gagan Singh Sonal, Madan Mohan Pradhan, Susanta K. Ghosh, Neena Valecha

**Affiliations:** 1 National Institute of Malaria Research, Indian Council of Medical Research, New Delhi, India.; 2 National Vector Borne Disease Control Programme, Delhi, India.; 3 National Vector Borne Diseases Control Programme, Bhubaneswar, India.; 4 National Institute of Malaria Research Field Unit, Bangalore, India.

## Abstract

Historically, malaria in India was predominantly caused by *Plasmodium vivax*, accounting for 53% of the estimated cases. After the spread of drug-resistant *Plasmodium falciparum* in the 1990s, the prevalence of the two species remained equivalent at the national level for a decade. By 2014, the proportion of *P. vivax* has decreased to 34% nationally, but with high regional variation. In 2014, *P. vivax* accounted for around 380,000 malaria cases in India; almost a sixth of all *P. vivax* cases reported globally. *Plasmodium vivax* has remained resistant to control measures, particularly in urban areas. Urban malaria is predominantly caused by *P. vivax* and is subject to outbreaks, often associated with increased mortality, and triggered by bursts of migration and construction. The epidemiology of *P. vivax* varies substantially within India, including multiple relapse phenotypes with varying latencies between primary infection and relapse. Moreover, the hypnozoite reservoir maintains transmission potential and enables reestablishment of the parasite in areas in which it was thought eradicated. The burden of malaria in India is complex because of the highly variable malaria eco-epidemiological profiles, transmission factors, and the presence of multiple *Plasmodium* species and *Anopheles* vectors. This review of *P. vivax* malaria in India describes epidemiological trends with particular attention to four states: Gujarat, Karnataka, Haryana, and Odisha.

## Background

**Figure 1.**
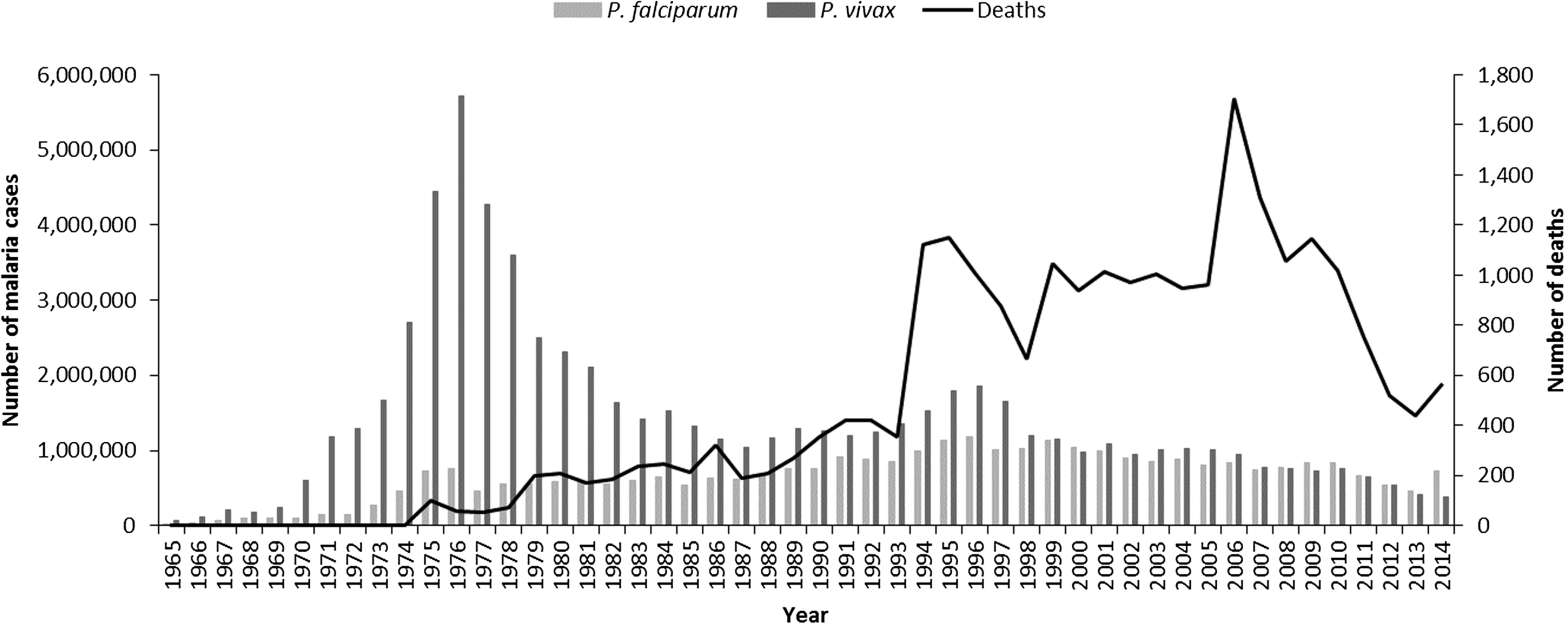
Annual malaria incidence in India, 1965–2014. Source: National Vector Borne Disease Control Programme.

India has by far the greatest estimated *Plasmodium vivax* burden of any country. In 2014, there were 2.14 million confirmed *P. vivax* cases globally, 18% of which occurred in India.^1^ *Plasmodium vivax* accounts for approximately a third of all malaria cases in India—with around 380,000 confirmed cases in the public sector in 2014 (Figure 1

). In the recent past, *P. vivax* accounted for around half of all malaria cases. Notably, *P. vivax* is an important pathogen in children. Around 30% of all *P. vivax* cases in India occur in children aged 1–14 years, though these represent just 12% of the total population.^2^

Malaria control measures in India include improved access to early diagnosis and treatment, the involvement of the accredited social health activists (ASHAs) at the community level, the introduction of artemisinin-based combination therapy, and intensified vector control, including long-lasting insecticidal nets (LLINs). Although malaria control measures have had impacts on both *Plasmodium falciparum* and *P. vivax* malaria, in most states, the recent declines in malaria cases and mortality have been achieved predominantly through the successful control of *P. falciparum* (Figure 1).^3^

**Figure 2.**
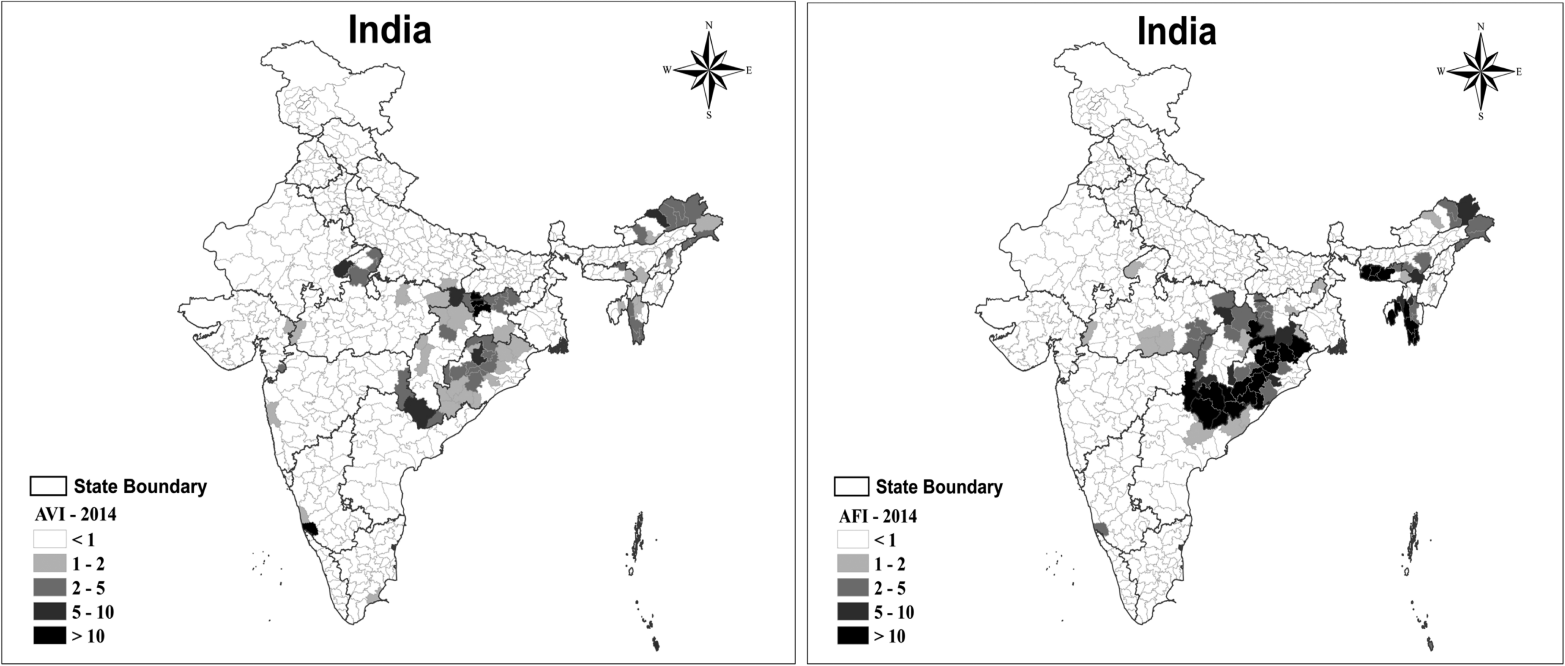
Annual parasite index (parasite incidence per 1,000 population) for *Plasmodium vivax* (AVI) and *Plasmodium falciparum* (AFI) in India, 2014. Source: National Vector Borne Disease Control Programme. The annual vivax index (AVI) is the ratio between the number of *P. vivax* cases reported and the population at risk per 1,000 inhabitants; a higher ratio indicates a more severe *P. vivax* malaria problem.

**Figure 3.**
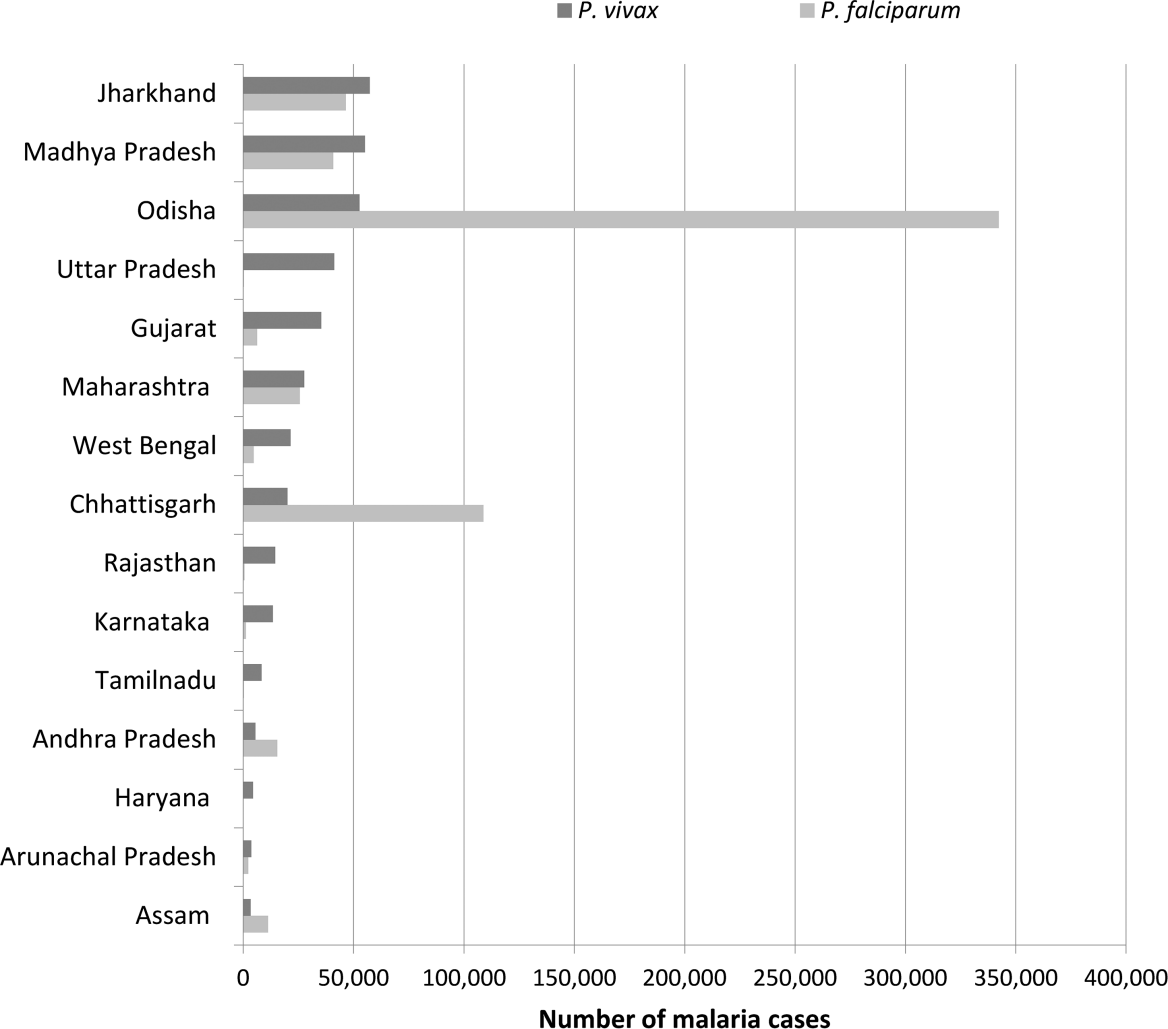
Indian states with more than 3,000 *Plasmodium vivax* malaria cases in 2014. Source: National Vector Borne Disease Control Programme.

The burden of malaria in India is complex and the proportions of *P. vivax* and *P. falciparum* vary across India (Figure 2

). Ten states account for around 89% of *P. vivax* malaria (Figure 3

), with 64% of cases arising in five states: Jharkhand, Madhya Pradesh, Odisha, Uttar Pradesh, and Gujarat.

**Figure 4.**
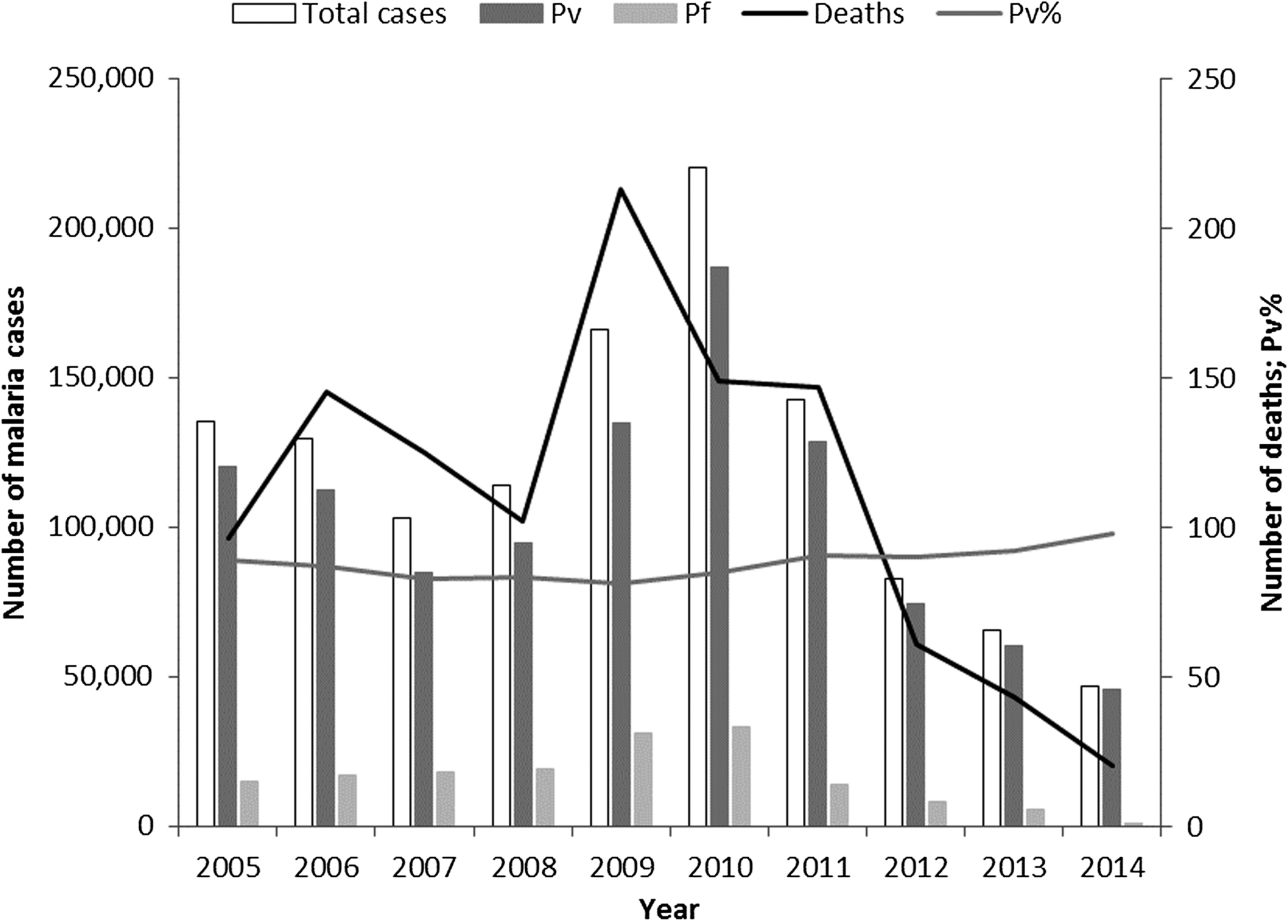
Urban malaria in India. Annual malaria incidence in the 19 states included in the Urban Malaria Scheme, 2005–2014. Source: National Vector Borne Disease Control Programme. Pf = *Plasmodium falciparum*; Pv = *Plasmodium vivax*; Pv% = percentage of total malaria attributed to *P. vivax*.

*Plasmodium vivax* is particularly troubling in the urban setting in India. Malaria control in such areas is problematic because of rapid construction, migration, and the mushrooming of slums. Because of the importance of urban malaria, these areas are covered by a special program—the Urban Malaria Scheme. In 2014, around 4% of all malaria cases, but 12% of all *P. vivax* cases in India occurred in urban areas. Within the Urban Malaria Scheme, 98% of all malaria cases were *P. vivax* in 2014 (Figure 4

). Urban areas are prone to malaria outbreaks and these are associated with peaks in mortality.

In this report, the malaria epidemiological situation for *P. vivax* is described for India, with examples taken from four states with differing eco-epidemiological situations: three that have a predominantly *P. vivax* burden (Gujarat, Karnataka, and Haryana) and one with a predominantly *P. falciparum* burden (Odisha).

## Burden of Relapse

As *P. vivax* relapse cannot be reliably differentiated from recrudescence or reinfection, reported *P. vivax* recurrence rates (including relapse, recrudescence, and reinfection) in India are given in Table 1 .^4^–^14^ *Plasmodium vivax* transmission occurs throughout the year, but is highest during the rainy and postrainy seasons, similar to the situation with *P. falciparum*. *Plasmodium vivax* appears earlier than *P. falciparum* because of its shorter incubation interval (21 days compared with 35 days, respectively) and transmission peaks before that of *P. falciparum*.

In Gujarat, about 28% of cases of malaria appear during the dry season, most probably caused by *P. vivax* relapses. Data from two studies conducted in Kheda district of Gujarat showed higher recurrence rates in the 5–10 years age group than in other age groups (Table 2 ).^12^,^13^ A total of 82% of recurrences occurred within 1 year of the primary malaria attack.^13^ Recurrences occurred up to 3 years after the primary malaria attack, but were less frequent in the 3rd and 4th year.^12^ Data from a semiarid region of Kutch in Gujarat showed significant autocorrelation peaks between the months of August to November and those from January to June, providing an estimated relapse latency period between 5 and 8 months and a mean value of 7 months. This relatively long interval is compatible with infection with temperate *P. vivax*.^15^

There is variation in relapse patterns both across and within states. In fact, strains with differing patterns of relapse can coexist, complicating transmission control measures. For example, in Delhi, *P. vivax* malaria populations are polymorphic for relapse: Group I (tropical type), the most common type, with relapse between 1 and 3 months; Group II, relapse between 3 and 5 months; and Group III (temperate type), relapse between 6 and 7 months.^4^,^5^ In contrast, in Mumbai the relapse pattern is predominantly of the tropical type.^6^

## Mosquito Vectors

Of the 58 *Anopheles* species in India, only six are epidemiologically important for malaria transmission with regional distribution, though other species may be key local vectors. Each species exhibits specific behavior and a preferred habitat. Multiple vector species may be present across any region, but no vector species is found throughout all of India.^16^

• *Anopheles culicifacies* s.l. is the main malaria vector in rural, peri-urban areas and in the plains, and is responsible for an estimated 65% of malaria in India. This species is mostly zoophagic and breeds in plain-land ecosystem.
• *Anopheles stephensi* is the key vector for malaria in urban areas, primarily zoophagic, but prefers human hosts in the absence of cattle. Curing waters in construction sites are a primary breeding area for this vector.
• *Anopheles dirus* s.l. is an efficient vector in the forest areas of northeastern regions. It is exophagic and exophilic and breeds in temporary water collections.
• *Anopheles minimus* is also a vector in the forest areas of northeastern regions, exhibiting zoophilic and exophilic behavior, breeding in slow-flowing streams.
• *Anopheles fluviatilis* s.l. is associated with hill and foothill areas, contributing 15% of malaria transmission in India.
• *Anopheles sundaicus* breeds in brackish water and is the main malaria vector in Andaman and Nicobar islands.

## Malaria Control Interventions

Malaria control policies are developed nationally via the National Vector Borne Disease Control Programme (NVBDCP). Implementation is at the state level, every state requiring a Vector Borne Disease Control Division.^17^ There are no *P. vivax*-specific vector control measures, but they are integrated with malaria control activities.

The key components of the Indian malaria control strategy for *P. vivax* and *P. falciparum* are as follows:

• Surveillance and case management
• Integrated vector control
• Epidemic preparedness and early response
• Supportive interventions, including behavior change communications and monitoring and evaluation

Vector control measures include insecticide-treated nets, LLINs, indoor residual spraying (IRS), antilarval measures, including fish and chemicals, and WHO Pesticide Evaluation Scheme-recommended biolarvicides, along with minor environmental engineering measures.

According to the national policy, all fever cases clinically suspected of being malaria should be confirmed by either microscopy or rapid diagnostic test (RDT). Prior to 2013, monovalent RDTs which detect PfHRPII/III were used in *P. falciparum* predominant areas. These only capture *P. falciparum* infections and not *P. vivax*. Blood slides of patients negative for *P. falciparum* using monovalent RDTs were sent for microscopy confirmation, at which point *P. vivax* would be detected, if present. In 2013, bivalent RDTs which detect and differentiate *P. falciparum* and *P. vivax* were introduced for use initially at lower level facilities and the community level, with scale-up in 2014. This new policy should provide a more accurate picture of the *P. vivax* burden, which may have been underestimated with the monovalent RDT, particularly in states where *P. falciparum* predominates, such as Odisha.

About 60% of blood tests are based on passive tests, that is, people reporting to the health facility. Passive case detection is highest during the monsoon rains (June–September), reflecting high malaria transmission. In *P. vivax* areas, a second, smaller peak occurs in March, possibly caused by *P. vivax* relapses. Diagnosis is usually obtained within a clinically relevant timeframe, even using microscopy. However, based on NVBDCP estimates, about 40% of malaria cases occur in difficult-to-reach areas, where microscopy results cannot be obtained within 24 hours.^18^

## Chemotherapy

Parasitologically confirmed *P. vivax* cases are given a 3-day course of chloroquine (25 mg/kg) to treat the acute blood stage infection plus a 14-day course of primaquine (0.25 mg/kg) to clear the dormant hypnozoite stage (radical cure). Primaquine is contraindicated in infants and pregnant women, and individuals with glucose-6-phosphate dehydrogenase (G6PD) deficiency. There is no G6PD deficiency testing in the field, but patients are instructed to stop primaquine treatment and report back to the clinic in cases of hematuria or dark urine, cyanosis or blue coloration of the lips. Antimalarial treatment is provided in blister packs, free of charge to all patients in the public sector according to the age-specific dosing schedule. Primaquine therapy is not directly observed and adherence data are lacking.

In urban areas, the private sector is an important provider of health services, but adherence to government prescribing guidelines is more limited in this sector.^19^–^21^ A study investigating the antimalarial prescribing habits of qualified physicians and pharmacists found that primaquine was prescribed to 87% of patients with confirmed *P. vivax* in the public sector versus 52% in private health facilities.^20^ For many poor communities using “less-than-fully-qualified” healthcare providers in the private sector, acute symptomatic treatment is the priority, and radical cure remains inaccessible.^19^ Until addressed, the lack of access to radical cure will continue to fuel the number of *P. vivax* malaria cases, first because several relapses can be caused by one infective bite and second because relapses maintain *P. vivax* transmission.

### Drug resistance.

Chloroquine remains safe and efficacious for the treatment of *P. vivax* malaria in India. *Plasmodium vivax* studies are also routinely conducted to track the emergence of chloroquine resistance in this species.^2^ There have been sporadic reports of chloroquine resistance in the country.^22^,^23^ However, therapeutic efficacy studies carried out at various sites could not confirm chloroquine resistance.^24^ Sentinel surveillance studies conducted among 185 *P. vivax* patients during 2009–2010 and 336 *P. vivax* patients during 2011–2012 showed 100% chloroquine efficacy.^25^,^26^

### G6PD deficiency.

G6PD deficiency testing is not carried out within the framework of the NVBDCP, neither is mass neonatal screening for G6PD deficiency conducted in India. Thus, data on G6PD deficiency prevalence is based on research studies. However, sample sizes have been generally small and sampling nonrepresentative of the population at risk of malaria. Data are insufficient to determine any relationship between the distribution of G6PD deficiency and the malaria burden either now or in the past to elucidate any epidemiologically relevant patterns. Operational research to estimate the prevalence of G6PD deficiency in the population has been included as an objective for the *P. vivax* elimination strategy as part of the National Framework for Malaria Elimination in India.^27^ This should provide evidence for the NVBDCP to assess whether and where to introduce G6PD testing before radical cure.

Pooling of data from 224 studies found a frequency of G6PD deficiency of 4.5%, varying from absent to 27.1% among the Angami Nagas tribe.^28^ A higher prevalence of G6PD deficiency (number evaluated) was observed in the east (6.7%, *N* = 2,899), central (6.1%, *N* = 329), and north (5.8%, *N* = 9,772) of India compared with the west (4.1%, *N* = 17,575) and south (3.2%, *N* = 8,908), where it is mainly confined to Andhra Pradesh and Tamil Nadu.^28^ For ethnic groups, the frequency was highest in scheduled tribes (5.5%, *N* = 5,468) and community (4.9%, *N* = 22,790) compared with caste (3.6%, *N* = 5,303) and scheduled castes (2.7%, *N* = 5,922).^28^ However, these proportions vary greatly depending on region, for example in north India the prevalence of G6PD deficiency is 6.6% in the caste group. Also, there are more than 4,500 different ethnic populations in India, and certain groups may have a particularly high prevalence of G6PD deficiency, for example 24.4% (*N* = 767) in the Kharia of Odisha.^29^

For the regions considered in more detail, the estimated prevalence of G6PD deficiency was 4.2% for Gujarat (*N* = 1,473), 2.0% for Haryana (*N* = 49), 0% for Karnataka (*N* = 87), and 1.1% for Odisha (*N* = 200).^28^ Given that in 2006 when this analysis was performed, the population of these regions exceeded 160 million, the lack of data on G6PD deficiency prevalence in India is clear.

Multiple G6PD genotypes exist in India and severe G6PD variants, such as G6PD*Mediterranean, are distributed throughout the country.^28^ As different variants confer different levels of risk for clinically important hemolysis, this complicates the evaluation of risk:benefit for both primaquine and potential G6PD diagnostic tests. However, there are few reports of acute hemolytic anemia,^24^ despite routine use of primaquine 0.25 mg/kg for 14 days following standard chloroquine therapy for *P. vivax*.^30^ Patient under-reporting of treatment complications, inadequate pharmacovigilance, as well as possible lack of compliance with the 14-day primaquine regimen could be explanations. Some healthcare providers are concerned about hemolytic side effects of primaquine and may be reluctant to prescribe. However, absence of radical cure treatment maintains *P. vivax* transmission via relapses and is refractive to elimination efforts. The availability of a suitable G6PD test would provide confidence in providing antirelapse therapy and improve access. Although two tests are commercially available, one is unsuitable for public health use in India because of cost ($15/test) and environmental restrictions (use above 25°C prohibited) and neither test is prequalified by the WHO.^31^

## Surveillance System

**Figure 5.**
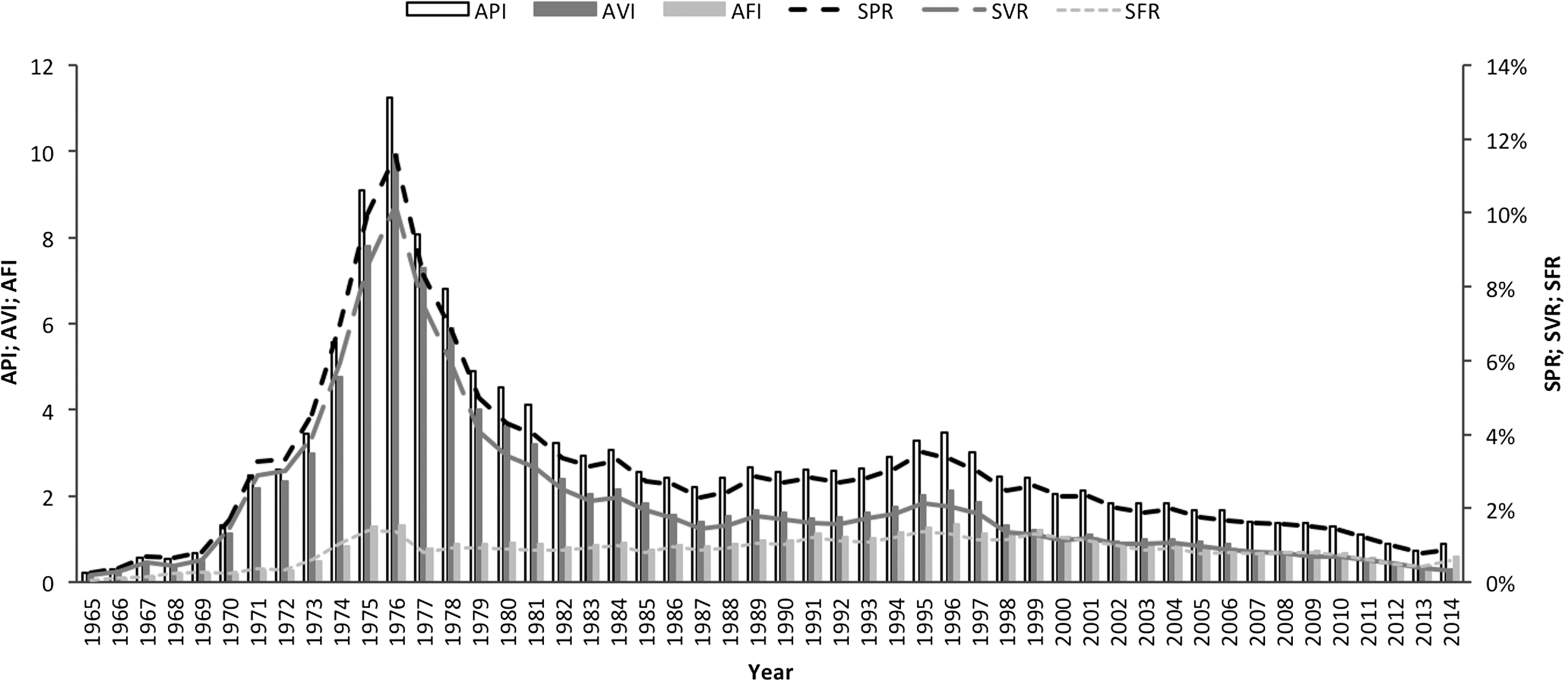
Changes in key indices of malaria epidemiology in India, 1965–2014. Source: National Vector Borne Disease Control Programme. API = annual parasite index; AVI = annual vivax index; AFI = annual falciparum index; SPR = slide positivity rate; SVR = slide positivity rate for *Plasmodium vivax*; SFR = slide positivity rate for *Plasmodium falciparum*.

India has a sound surveillance system and data are consistently reported.^17^ Over 100 million blood slides are examined in India every year, providing a rich source of surveillance data from all endemic districts. Between 2001 and 2014, the annual blood examination rate has remained fairly constant at about 10%. The annual parasite index (number of confirmed malaria cases per 1,000 population) has consistently declined from 2.12 per 1,000 in 2001 to 0.89 in 2014. The slide positivity rate (% of slides positive) has also reduced over this period from 2.31% in 2001 to 0.89% in 2014. The slide positivity rate was higher in *P. falciparum* (0.59) than in *P. vivax* (0.3) in 2014 (Figure 5

).

Three key methods of surveillance are used:

• Active case detection is carried out in rural areas; blood smears of people with fever or who recently had fever are collected by multipurpose workers during fortnightly house visits.
• Passive case detection is carried out in individuals with fever who report to peripheral health volunteers or ASHA, at subcenters by RDTs and at primary health facilities.
• Mass survey of the entire population is carried out during an epidemic. Blood smears are taken from the entire population irrespective of age, sex, or fever status.

Manually aggregated line lists for each person are entered into a database. Villages are stratified based on the overall annual parasite index. The incidence of *P. vivax* malaria is not a criterion for stratification. A key limitation is that mixed infections are recorded as *P. falciparum* malaria in aggregated reports and mixed infections are often likely to be missed in microscopy. *Plasmodium vivax* malaria deaths are investigated and recorded; however, these are not included in the annual report.

## Epidemiological Trends by Selected States

**Figure 6.**
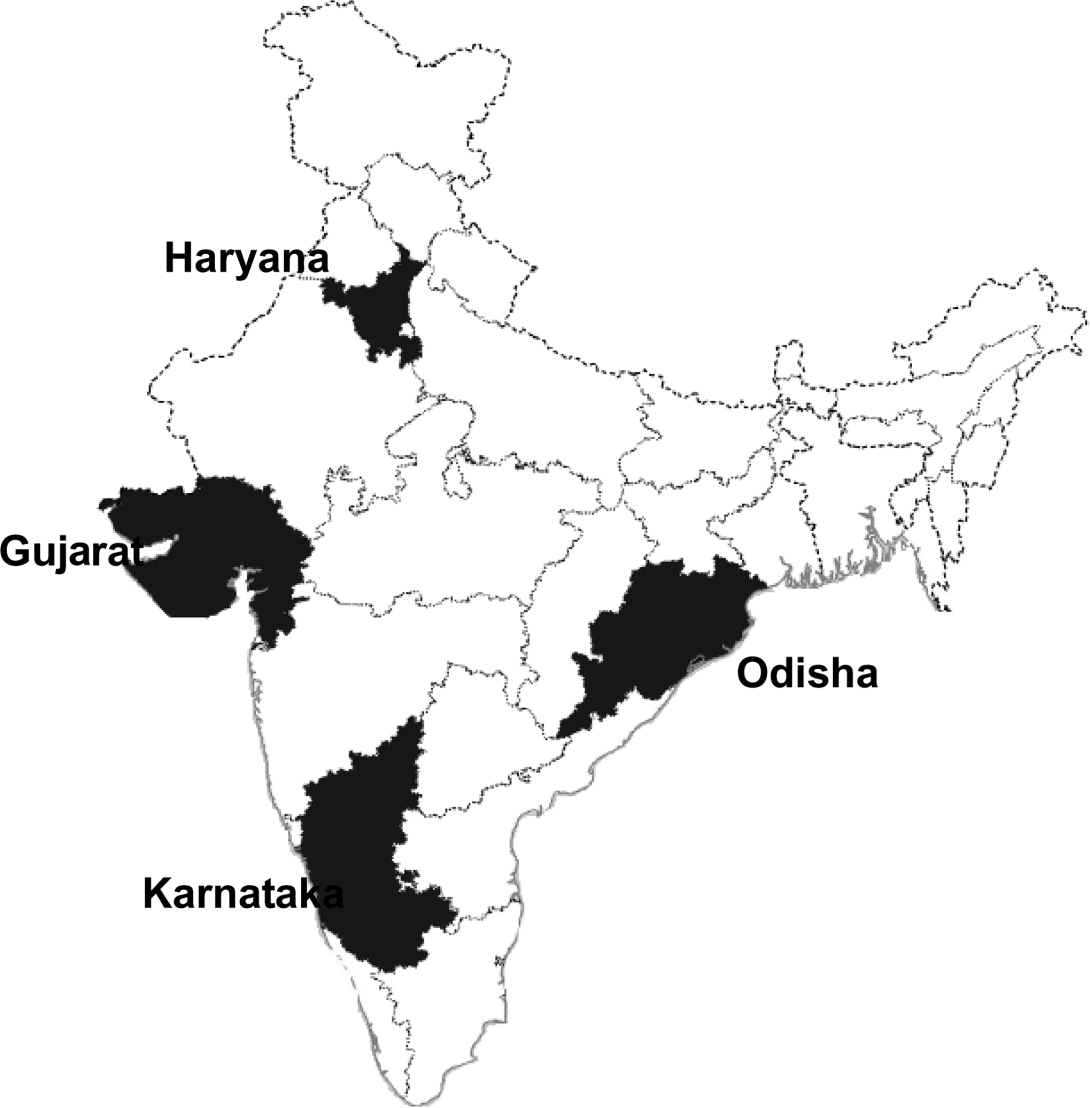
Map of India showing the states considered in detail.

In view of extensive regional diversity, this review on *P. vivax* malaria in India explores the situation at both the national level and in four states from four regions in the country: northwest (Gujarat), north (Haryana), east (Odisha), and southwest (Karnataka) (Figure 6

). Each of these states has a well-developed public health services, with reliable surveillance systems. Overall, the four states accounted for 29.1% of the nationally reported 364,611 *P. vivax* cases in 2014. Further, there is diversity among these states in terms of *Plasmodium* species, endemicity, transmission patterns, etc. Notably, there appears to be a key trend across Gujarat, Odisha, and Karnataka for an increase in the proportion of malaria cases attributable to *P. vivax* as the overall number of malaria cases declines.

### Gujarat.

**Figure 7.**
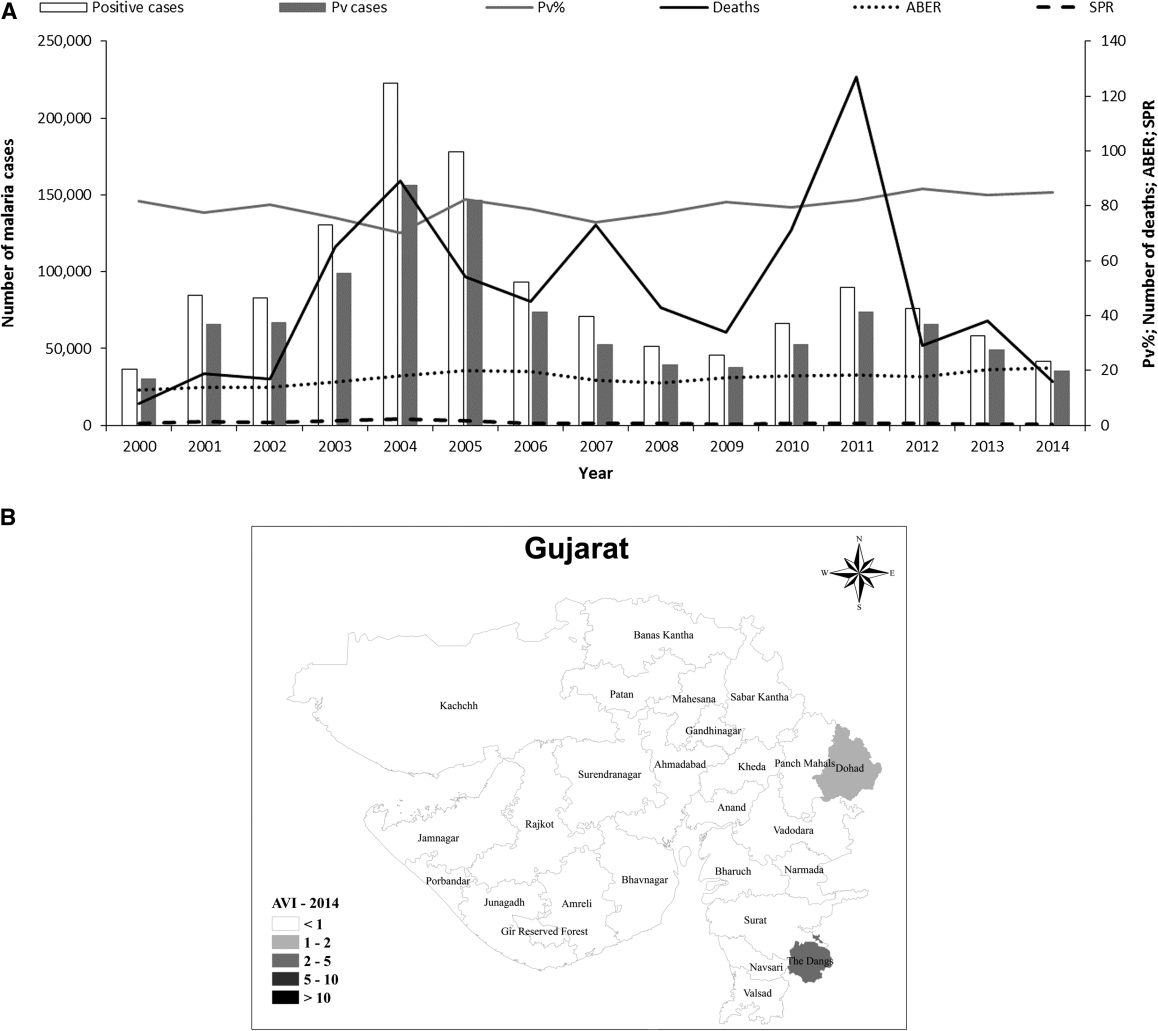
Malaria epidemiology in Gujarat. Source: State National Vector Borne Disease Control Programme. (**A**) Annual malaria cases and deaths in Gujarat, 2001–2014. Pv = *Plasmodium vivax*; Pv% = percentage of total malaria attributed to *P. vivax*; ABER = annual blood examination rate; SPR = slide positivity rate. (**B**) Annual *P. vivax* index (AVI) and *Plasmodium falciparum* (AFI) in Gujarat, 2014.

Gujarat, in northwestern India, is home to over 60 million people. It has a diverse climate, with desert regions in the northwest of the state, whereas the southern districts are wetter because of the monsoon, which starts around mid-June. Gujarat has the third highest *P. vivax* malaria burden in the country, accounting for 9% of the national total. In normal years, the *P. vivax*/*P. falciparum* proportion in Gujarat is about 80:20. However, the *P. falciparum* proportion increased from 18% to 30% during two major malaria outbreaks in 2004 and 2011 (Figure 7A

). Higher rates of *P. falciparum* infection were generally associated with an increase in the proportion of severe malaria cases and deaths from malaria. However, leaving aside the increased mortality associated with epidemics, there has been an overall decline in the number of malaria deaths in Gujarat. The highest number of deaths was recorded in 2011 (127 deaths) (Figure 7A). Despite extensive investigations, no clear cause has been identified the high mortality rate in 2011.

After malaria outbreaks, *P. falciparum* appears to be easier to control than *P. vivax*. Intensified malaria control efforts, in particular vector control, has led to a decline in the number of malaria cases, particularly those caused by *P. falciparum* (Figure 7A). The epidemiological impact of *P. falciparum* outbreaks is limited, with a median change in incidence of −13% in the year after the outbreak. In contrast, *P. vivax* outbreaks result in a longer setback, with a + 30% median change in incidence in the year postoutbreak (N. Shah, personal communication). This is probably because of the impact of *P. vivax* relapses; both in terms of cases of clinical malaria and because relapses generate gametocytes, thus maintaining transmission. After the setback of 2011, there has been a dramatic improvement in the overall incidence of *P. vivax* in Gujarat on a district level, following the trend for India overall. The wider introduction of the bivalent RDT may also have contributed to post-2011 declines. However, six districts still had an annual vivax incidence of one or more in 2014 (Figure 7B).

### Haryana.

**Figure 8.**
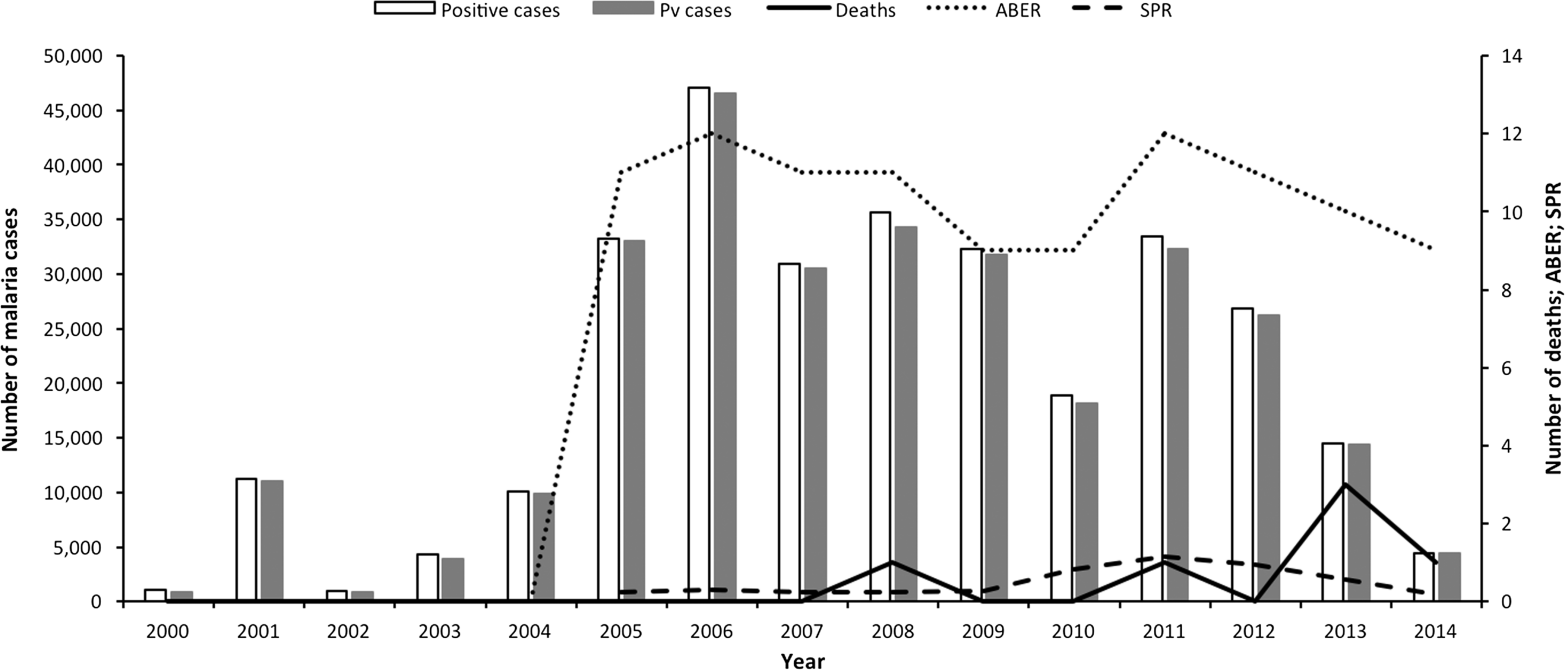
Malaria epidemiology in Haryana: trends in annual malaria incidence in Haryana, 2000–2014. Pv = *Plasmodium vivax*; ABER = annual blood examination rate; SPR = slide positivity rate. Source: State National Vector Borne Disease Control Programme.

In the far north of India, Haryana is a key agricultural state, dependent on the monsoon rains with a population of over 25 million people. Virtually, all malaria cases are caused by *P. vivax* in this region. The *P. vivax* burden in Haryana has been dramatically reduced from 46,571 cases in 2006 to 18,158 in 2010 only to increase to 32,268 in 2011 following very heavy rainfall (Figure 8

).^32^ This greatly expanded the range and duration of mosquito breeding grounds and was reflected in an increase in slide positivity rate, indicating increased malaria transmission. The caseload has since declined steadily to only 4,440 cases of malaria in 2014. This decline is probably because of effective vector control activities, including: IRS, fogging during outbreaks, and space spraying during the day; extensive entomological investigations to find breeding sites so that these can be removed, modified, or treated with larvicides; the use of larvivorous fish; and public education on personal measures for protection (bed nets, repellents, covering bare skin, and house screening) and for reducing breeding sites around the home. By 2014, there was no district with an annual vivax incidence of more than one. The state accounts now for about 1% of the total national *P. vivax* malaria burden as against 5.1% in 2006.

### Karnataka.

**Figure 9.**
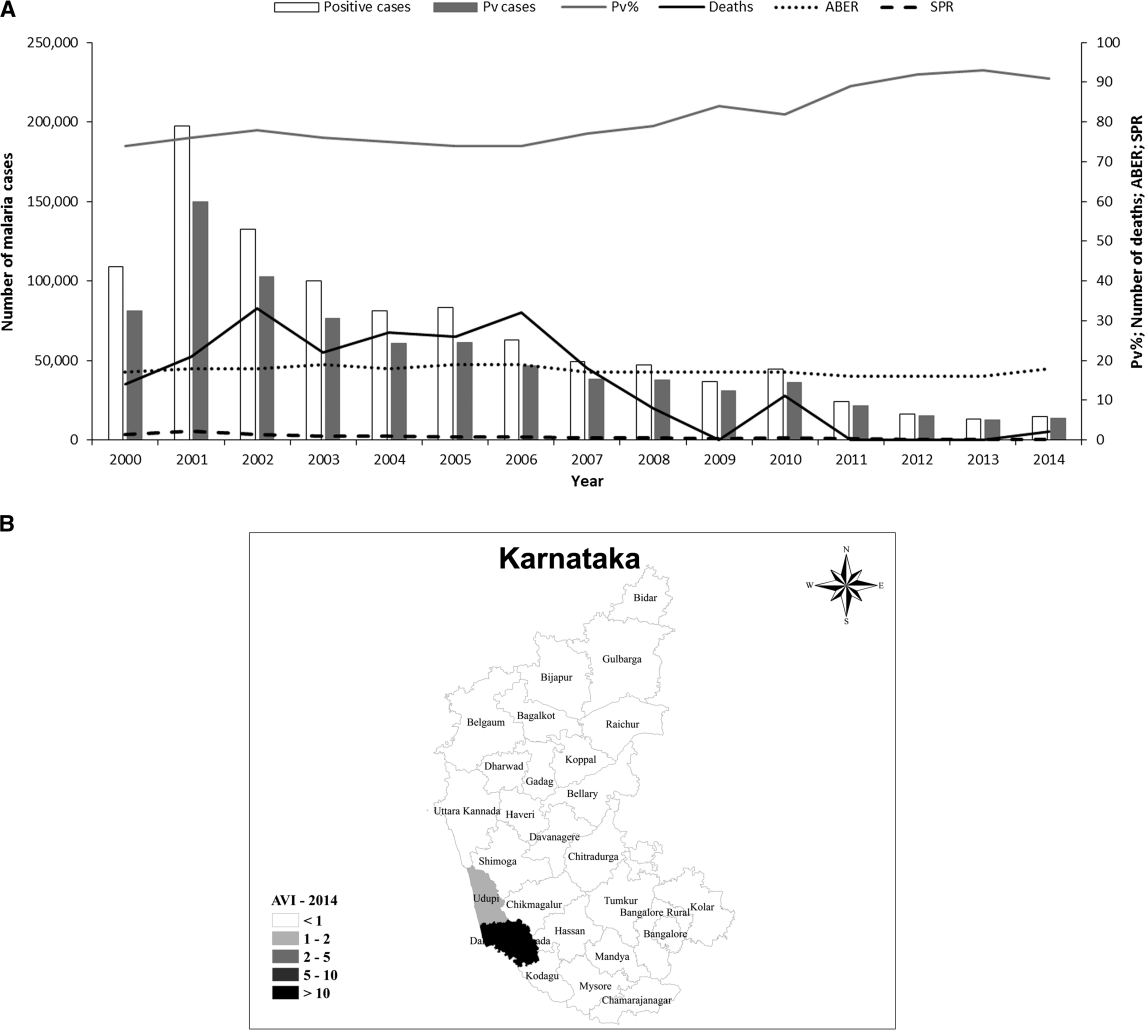
Malaria epidemiology in Karnataka. Source: State National Vector Borne Disease Control Programme. (**A**) Trends in annual malaria incidence in Karnataka, 2000–2014. Pv = *Plasmodium vivax*; Pv% = percentage of total malaria attributed to *P. vivax*; ABER = annual blood examination rate; SPR = slide positivity rate. (**B**) Annual *P. vivax* index (AVI) and *Plasmodium falciparum* (AFI) in Karnataka, 2014.

In southwestern India, Karnataka has a population of around 64 million people and accounts for 3% of India's total *P. vivax* malaria burden. Most of the population is involved in agriculture and the climate varies from rather wet in the Malnad and coastal regions to the arid Deccan Plateau in the north of the state. There was a dramatic increase in the number of cases of malaria at the start of the decade, mainly due to malaria outbreaks in northern districts. Subsequently, surveillance was intensified in endemic districts and larvivorous fish were also released. The situation has since improved substantially, with an 81% reduction in *P. vivax* malaria cases and a 95% reduction in those caused by *P. falciparum*.^33^ The *P. vivax* malaria proportion increased from 74% at the start of the decade to 91% in 2014. The very low slide positivity rate reflects the very low transmission with a constant annual blood examination rate and zero malaria-related deaths in the state (Figure 9A

). In Karnataka, malaria mortality has always been low. Official figures recorded no deaths in 2011–2013 and only a total of 21 deaths between 2008 and 2014 (Figure 9A). The overall reduction in malaria burden is also reflected at a district level; most districts have an annual vivax index of < 1. Dakshin Kannada (Mangalore) district of Karnataka has the highest annual vivax index of < 10 (Figure 9B) (see also section on urban malaria).

### Odisha.

**Figure 10.**
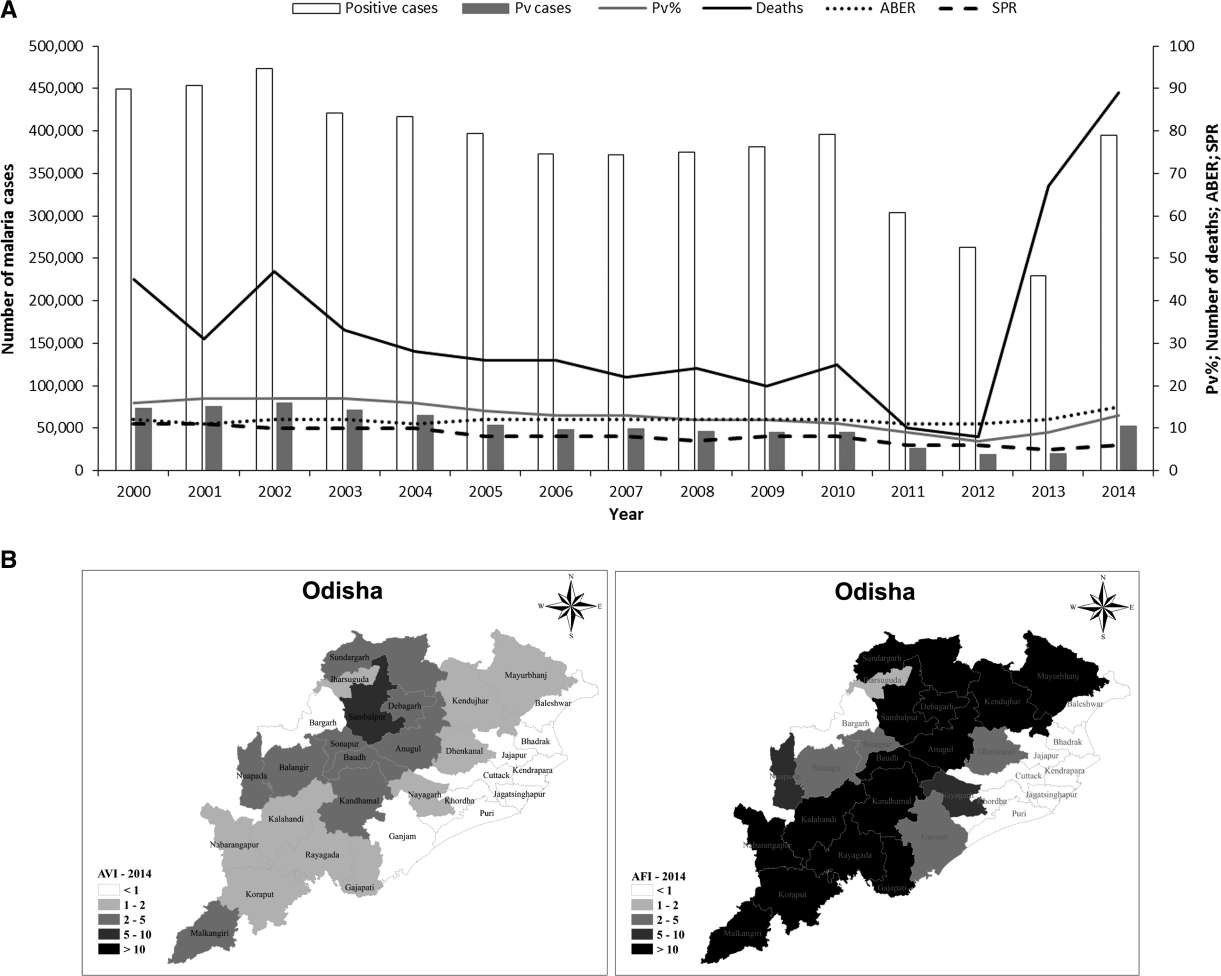
Malaria epidemiology in Odisha. Source: State National Vector Borne Disease Control Programme. (**A**) Trends in annual malaria incidence in Odisha, 2000–2014. Pv = *Plasmodium vivax*; Pv% = percentage of total malaria attributed to *P. vivax*; ABER = annual blood examination rate; SPR = slide positivity rate. (**B**) Annual parasite index (parasite incidence per 1,000 population) for *P. vivax* (AVI) and *Plasmodium falciparum* (AFI) in Odisha, 2014.

On the eastern coast, Odisha is in the tropical coastal region, with monsoon rains July to September and less severe rains in October and November with the retreating monsoon. Odisha is a center for intense rice cultivation in the coastal alluvial plains. Odisha is the most malaria endemic state in India, accounting for a quarter of all cases. Although only 3% of the population live in Odisha, 47% of all *P. falciparum* cases reported in India occur here. *Plasmodium vivax* accounts for 13% of malaria cases in Odisha (about 53,000 cases in 2014). There had been a dramatic improvement in the malaria situation in Odisha from 2010 to 2013, with a 41% reduction in cases and an 86% reduction in deaths, with a relatively constant annual blood examination rate. In 2014, the malaria burden for both *P. falciparum* and *P. vivax* went back to the 2010 levels because of a number of factors, including improved surveillance, and the introduction and scale-up of bivalent RDTs (Figure 10A

). This trend was also observed in other malaria endemic states. There has been a slight increase in the proportion *P. vivax* cases from 11% in 2010 to 13% in 2014. The slide positivity rate remains high, reflecting the high transmission of *P. falciparum* malaria in the state.

The incidence of *P. vivax* malaria has increased in all districts. In 2014, 10 districts had an annual vivax index of 1–2, nine an annual vivax index of 2–5, and one an annual vivax incidence of 5–10. In contrast, *P. falciparum* malaria remains problematic across the state (Figure 10B).

## The Unique Problem of Urban Malaria

In recent years, urban malaria has become an important challenge for malaria control in India. The Urban Malaria Scheme is managed by the municipalities under guidance from the NVBDCP. *Anopheles stephensi* is the principal vector responsible for transmission of urban malaria, breeding in the numerous overhead tanks, construction sites, etc. typical of Indian cities. The high and steady migration of people from malaria endemic areas maintains the parasite burden. Furthermore, there had been reports of resistance to the commonly used insecticides in many places in India.^34^ Larval source management is recommended to control the urban malaria vector *An. stephensi*.

### Gujarat.

**Figure 11.**
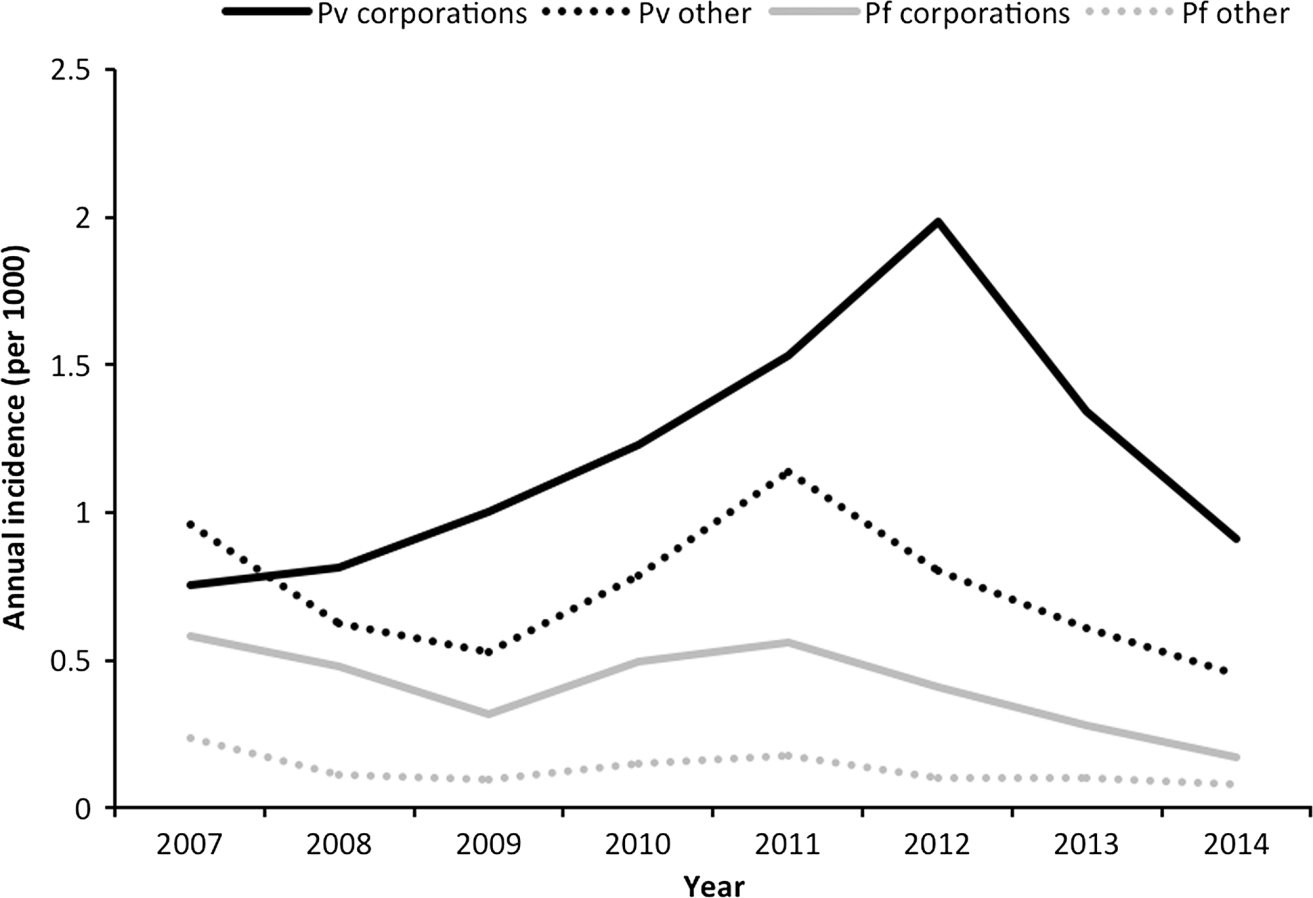
Annual *Plasmodium vivax* and *Plasmodium falciparum* malaria incidence by urban and nonurban areas in Gujarat 2007–2014. Source: State National Vector Borne Disease Control Programme. Pf = *P. falciparum*; Pv = *P. vivax*.

In Gujarat, about 40% of all malaria cases were reported from eight corporations and 12 towns under the Urban Malaria Scheme, which covers 24% of the state population. The annual incidence of *P. vivax* malaria in urban areas was 2.5 times greater than in nonurban areas, where *P. vivax* appears to be more responsive to control measures (Figure 11

). Annual blood examination rates are high in both urban and nonurban areas. A study in Ahmedabad found that, although malaria was found to be spatially heterogeneous within the city, it was temporally stable. Thus, data from previous years could be used to predict malaria burden and indicate where targeted control measures should be implemented.^35^

### Haryana.

There are 17 towns with more than 50,000 population in which the Urban Malaria Scheme is being implemented in Haryana. The National Capital Territory of Delhi is surrounded by urban areas which project into the neighboring states of Haryana, Uttar Pradesh, and Rajasthan. Thus, there is an overlap between the urban malaria issue in Haryana and that of the capital.

In Haryana, urban malaria contributes about 12% of all *P. vivax* malaria cases in the state and *P. vivax* is responsible for most of the malaria cases recorded under the Urban Malaria Scheme in this state.^32^ However, urban malaria may be underestimated in the slum areas around Delhi, as many families rely on local private health practitioners and malaria prevalence data is not collected in the private healthcare setting.^19^

### Karnataka.

Urban malaria is a major problem in Karnataka and the coastal city, Mangalore, accounted for 57% of malaria cases in 2014 compared with only 3% in 2002. *Anopheles stephensi* is the main vector, breeding in wells, fountains, cement tanks, and other standing water. Bellary town also has a significant malaria problem. This shift from rural to urban malaria was caused by drastic reductions in rural malaria cases after sustained vector control activities with larvivorous fish coupled with early diagnosis and prompt treatment.^36^

### Odisha.

In Odisha, the main malaria burden is centered on the forested areas in the tribal regions of the southern area of the state. Urban malaria is, therefore, not such an issue in this state at present.^37^

## Epidemic Vivax Malaria

*Plasmodium vivax* malaria epidemics have been recorded in many states. The main reasons for malaria epidemics are identified as an inadequacy in surveillance, and insufficient IRS in rural areas, or antilarval measures in urban areas.^2^

### Gujarat.

The varied ecotypes in Gujarat make parts of the state prone to malaria outbreaks. From a peak in 2010, when 24% of districts were affected, there has been a declining trend in the number of outbreaks, with 15% of districts affected in 2011 and none thereafter. Most outbreaks in Gujarat are caused by *P. vivax* or mixed infections and are associated with higher mortality. In Gujarat, in 2011 there were 127 deaths, with 17.9% of these being *P. vivax* cases.^38^

As Gujarat is an industrialized and urban area, migration from rural areas, including seasonal workers living in temporary accommodation, is common. Malaria control efforts are mainly targeted on stable rural and urban populations, so influxes of migratory workers can lead to uncontrolled reservoirs of malaria that can spill over into the resident population. Such outbreaks place considerable pressure on local healthcare efforts.^39^

### Haryana.

There have been no malaria outbreaks in Haryana in the recent past.

### Karnataka.

In the past, *An. fluviatilis* was the predominant vector involved in malaria outbreaks. However, deforestation and the extensive use of insecticides has caused this species to disappear from Karnataka.^33^ Subsequent invasion by *An. culicifacies* has led to this species now being the most important vector leading to outbreaks in rural/semiurban areas.^40^ In the urban areas, *An. stephensi* is the key vector. In outbreaks, generally, *P. vivax* cases far exceed those of *P. falciparum*.

### Odisha.

Odisha is a malaria endemic state, with perennial transmission. However, the coastal belts of the state show low malaria incidence (< 2 annual parasite index), and outbreaks can occur in these areas when conditions increase local environmental receptivity to human transmission. *Anopheles culicifacies*, *An. Fluviatilis*, and *An. annularis* are the three major vectors in the state, with *An. subpictus* a local vector.^41^,^42^ In 2010, a malaria outbreak occurred in Balasore, a coastal district of Odisha.^43^ The annual parasite index between 2007 and 2009 was 0.8, but 123 malaria cases were confirmed in 2010, in a population of just 839, though only six cases were positive for *P. vivax*.^43^ *Anopheles culicifacies*, *An. Annularis*, and *An. subpictus* were involved in transmission during the outbreak, which was thought to have been triggered by 3 days of heavy rain which filled five ponds in the area, followed by high temperatures (42°C), plus a low density of cattle in the area, leaving humans as the preferred food source.^43^

## Conclusions

*Plasmodium vivax* remains a substantial health and economic burden in India, and has proved difficult to control, particularly in urban areas. Even though malaria cases have declined in the recent past, the relative proportion of *P. vivax* cases are increasing. *Plasmodium vivax* shows polymorphism in the patterns of relapse, it is transmitted by a variety of vectors across diverse ecological habitats, and may be overlooked as a pathogen where a mixed infection with *P. falciparum* is present. There is increasing evidence that *P. vivax* is associated with severe malaria and cerebral malaria in India.^44^–^46^ This may be because of improved reporting and investigation and/or changes in *P. vivax* pathogenicity, which may be specific to individual parasite populations.^47^–^49^

The limitations of this study should be recognized in that only confirmed malaria cases treated in the public sector are considered. There are undoubtedly many malaria cases that are unreported or are treated in the private sector, and even malaria-related deaths which are attributed to other causes.^50^–^55^ However, the four states considered have well-developed and reliable reporting systems which allow comparison over time and between regions. There is likely to have been under-diagnosis of *P. vivax* before 2013, because of the use of monovalent RDTs. However, the overall trends before this date and following the introduction of bivalent RDTs are comparable.

Improvements in urban malaria control are needed, including the possibility of using IRS in urban areas. Further, peri-urban areas including slums are foci of *P. vivax* transmission and need to be targeted with treatment and control measures, especially regarding the accessibility of radical cure. In particular, the development of a validated, operationally feasible point-of-care G6PD test would ensure safe antirelapse therapy, thus preventing relapses and interrupting transmission. Extended access to quality bivalent RDTs and microscopy are needed to quickly identify outbreaks and improve surveillance with the aim of diagnosing and treating more cases.

Addressing the *P. vivax* burden is crucial for India to eliminate malaria, as the hypnozoite reservoir maintains the disease in areas where transmission is very low or very seasonal. In such areas, vector control measures can only achieve limited success, because opportunities for transmission, such as earlier or greater rainfall than expected, can be rapidly exploited by the parasite. Moreover, *P. vivax* malaria epidemics result in a longer setback for overall malaria control efforts because of the infection reservoir in the liver. In such a large and populous country, the challenges are great, but the benefits of reducing or even eliminating malaria will be even greater.

## Figures and Tables

**Table 1:** Relapse rates among patients with *Plasmodium vivax* malaria in India

Site	Year	Treatment	*n*	Follow-up (months)	% Relapse	Reference
Delhi	1988	CQ + PQ (5)	316	60	44.3	4
	1989	CQ + PQ (5)	487	48	30.2
	1990	CQ + PQ (5)	497	36	26.6
	1991	CQ + PQ (5)	524	24	28.4
	1992	CQ + PQ (5)	669	12	23.3
	2001	CQ + BQ	219	12	29.68	5
		CQ + PQ (5)	220	12	26.82
		CQ	224	12	40.18
Mumbai	1999	CQ		6	11.7	6
		CQ + PQ (5)		6	26.7
		CQ + PQ (14)		6	0
Mumbai	2003	CQ + PQ (14)	131	21	4.60	7
		CQ	142	21	9.20
Odisha	2002	CQ	723	12	8.6	8
		CQ + PQ (5)	759	12	6.5
Hardwar	1989	CQ + PQ (5)	725	13	6.9	9
	2001	CQ + PQ (5)	5,541	12	0.1–9.2	10
Mandla	1990	CQ + PQ (5)	995	8	10.3	11
Kheda, Gujarat	1990	CQ + PQ (5)	1,520	12	2.6	12
		CQ	264	12	18.9	
	1996	CQ	226	12	28.3	13
		CQ + PQ (5)	173	12	5.78	
		CQ + PY	136	12	27.7	
Shahjahpur	1986–1989	CQ + PQ (5)	13,720	48	2.03–23.17	14

BQ = bulaquine; CQ = chloroquine; PQ = primaquine; PY = pyrimethamine. Numbers in brackets indicate number of days of primaquine therapy.

**Table 2:** Malaria relapse risk in Gujarat^13^ (*N* = 262 patients with weekly follow-up)

Age range (years)	Recurrence (%)	Relative risk	Recurrences in cases with ≥ 1 recurrence	
			No. of recurrences	Patients *n* (%)
2–4	21.4	1.6	–	–
5–10	32.0	2.4	3	2 (2.0)
11–14	24.5	1.9	2	17 (16.7)
15–30	17.6	1.3	1	83 (81.4)
> 30	13.2	–	Total	102 (100)
